# Supplementary material for: COVID-19 pandemic and trends in new diagnosis of atrial fibrillation: A nationwide analysis of claims data
Source: PLoS One. 2023 Feb 2;18(2):e0281068. doi: 10.1371/journal.pone.0281068 (PMC9894497; doi:10.1371/journal.pone.0281068)
Supplement: S1 Table — The estimated level change shows the immediate change in the outcome following the World Health Organization declaration of pandemic on 3/11/2020. The estimated trend change depicts the further change from the predicted every 30 days (slope). (PDF) [file pone.0281068.s001.pdf]

Parameter	New Atrial Fibrillation Diagnoses, per 1000 Individuals		Ischemic Stroke as Initial Manifestation of Atrial Fibrillation, per 1000 Individuals		New Atrial Fibrillation Diagnoses: Inpatient Setting, per 1000 Individuals		New Atrial Fibrillation Diagnoses: Outpatient Setting, per 1000 Individuals	
	**Estimate**	***p*-Value**	**Estimate**	***p*-Value**	**Estimate**	***p*-Value**	**Estimate**	***p*-Value**
Level Change after 03/11/2020	-0.522	<0.001	-0.017	<0.001	-0.114	<0.001	-0.361	<0.001
Trend Change after 03/11/2020	0.096	<0.001	0.003	0.001	0.025	<0.001	0.063	<0.001
