# Supplementary material for: COVID-19 pandemic and trends in new diagnosis of atrial fibrillation: A nationwide analysis of claims data
Source: PLoS One. 2023 Feb 2;18(2):e0281068. doi: 10.1371/journal.pone.0281068 (PMC9894497; doi:10.1371/journal.pone.0281068)
Supplement: S2 Table — The estimated level change shows the immediate change in the outcome following the World Health Organization declaration of pandemic on 3/11/2020. The estimated trend change shows the further change from the predicted every 30 days (slope). (PDF) [file pone.0281068.s002.pdf]

Parameter	New Atrial Fibrillation Diagnoses in White Individuals, per 1000 Individuals		New Atrial Fibrillation Diagnoses in Black Individuals, per 1000 Individuals		New Atrial Fibrillation Diagnoses in Hispanic Individuals, per 1000 Individuals		New Atrial Fibrillation Diagnoses in Individuals of Other Races/Ethnicities, per 1000 Individuals	
	**Estimate**	***p*-Value**	**Estimate**	***p*-Value**	**Estimate**	***p*-Value**	**Estimate**	***p*-Value**
Level Change after 03/11/2020	-0.502	<0.001	-0.367	<0.001	-0.283	0.009	-0.536	<0.001
Trend Change after 03/11/2020	0.092	<0.001	0.072	0.001	0.040	0.16	0.096	<0.001
