# Supplementary material for: COVID-19 pandemic and trends in new diagnosis of atrial fibrillation: A nationwide analysis of claims data
Source: PLoS One. 2023 Feb 2;18(2):e0281068. doi: 10.1371/journal.pone.0281068 (PMC9894497; doi:10.1371/journal.pone.0281068)
Supplement: S3 Table — The linear regression adjusted for autocorrelation using Newey-West standard error correction. Analysis was conducted using PROC MODEL with the SAS/ETS software. A joint Wald test shows no statistically significant difference in the change in the level of AF diagnoses after pandemic declaration across racial/ethnic subgroups (p = 0.34). (PDF) [file pone.0281068.s003.pdf]

Parameter	New Atrial Fibrillation Diagnoses in White Individuals, per 1000 Individuals	
	Estimate	p-Value
Intercept	1.141	<0.001
Baseline trend	-0.001	0.55
Level change after 3/11/2020	-0.391	<0.001
Trend change after 3/11/2020	0.063	<0.001
Baseline level change for Black individuals	-0.134	<0.001
Baseline level change for Hispanic individuals	-0.413	<0.001
Baseline level change for individuals of other races/ethnicities	-0.199	<0.001
Baseline trend change for Black individuals	0.002	0.05
Baseline trend change for Hispanic individuals	0.001	0.33
Baseline trend change for individuals of other races/ethnicities	0.001	0.11
Level change after 3/11/2020 for Black individuals	0.003	0.96
Level change after 3/11/2020 for Hispanic individuals	0.063	0.19
Level change after 3/11/2020 for individuals of other races/ethnicities	-0.001	0.98
Trend change after 3/11/2020 for Black individuals	-0.006	0.57
Trend change after 3/11/2020 for Hispanic individuals	-0.015	0.13
Trend change after 3/11/2020 for individuals of other races/ethnicities	-0.013	0.04
