# Supplementary figures and images for: COVID-19 pandemic and trends in new diagnosis of atrial fibrillation: A nationwide analysis of claims data
Source: PLoS One. 2023 Feb 2;18(2):e0281068. doi: 10.1371/journal.pone.0281068 (PMC9894497; doi:10.1371/journal.pone.0281068)

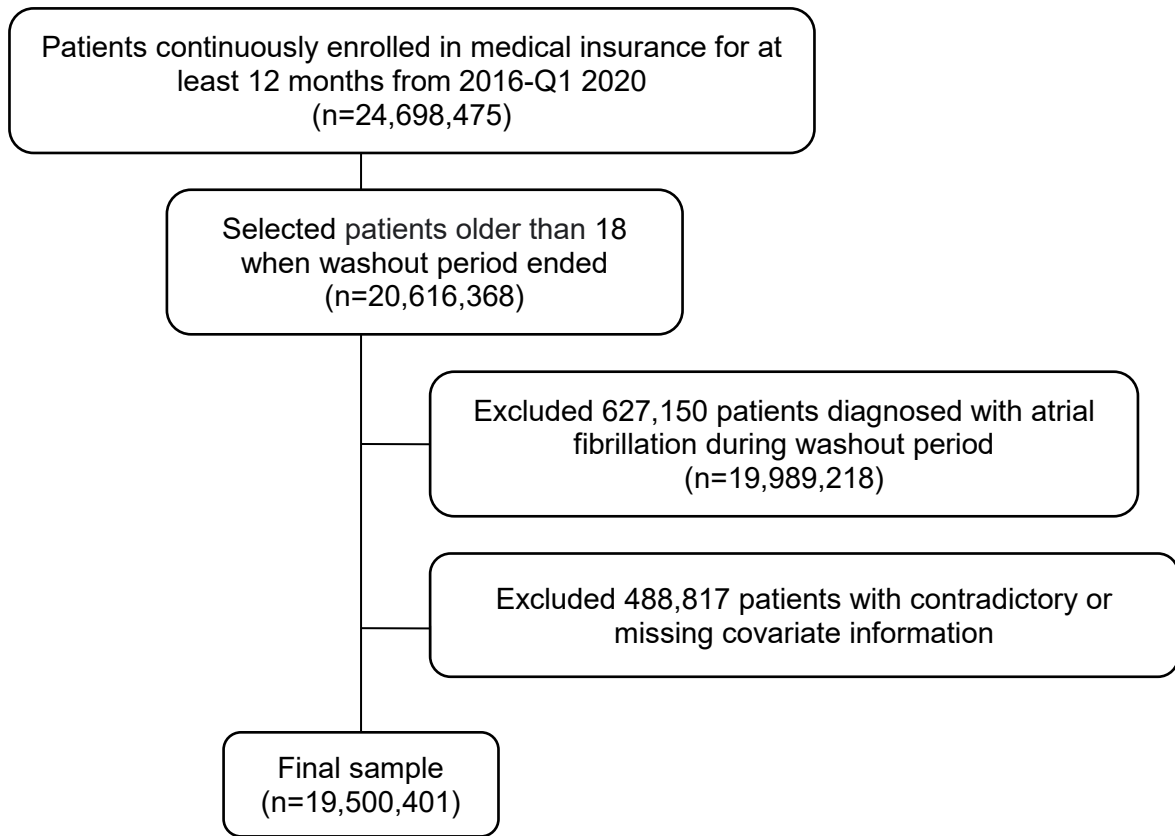

Supplement: S1 Fig — The study cohort was selected using 2016—Q3 2020 data. Atrial fibrillation was defined as having a diagnosis claim with the ICD-9 code of 427.31 or ICD-10 codes of I48.0, I48.1, I48.2, or I48.91 in the first or second diagnosis field. (PDF) [file pone.0281068.s005.pdf]

— observed value    — predicted value    - - - UCL    - - - LCL

### Female Individuals

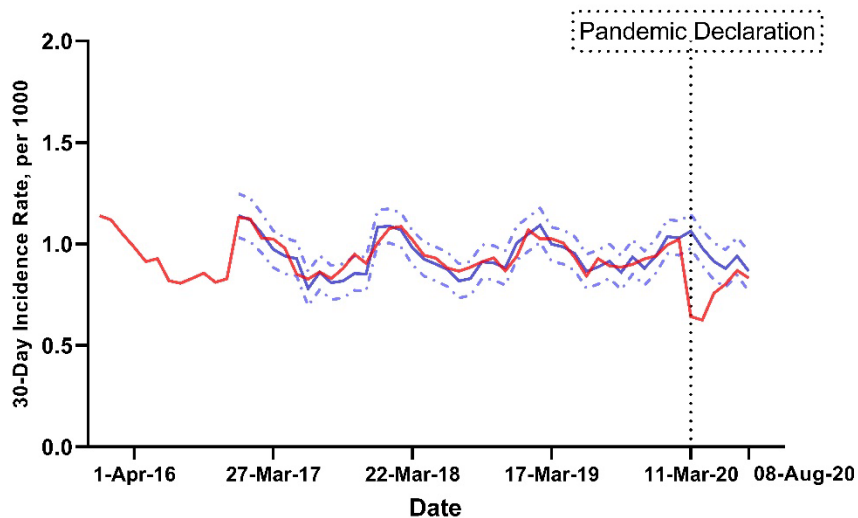

### Male Individuals

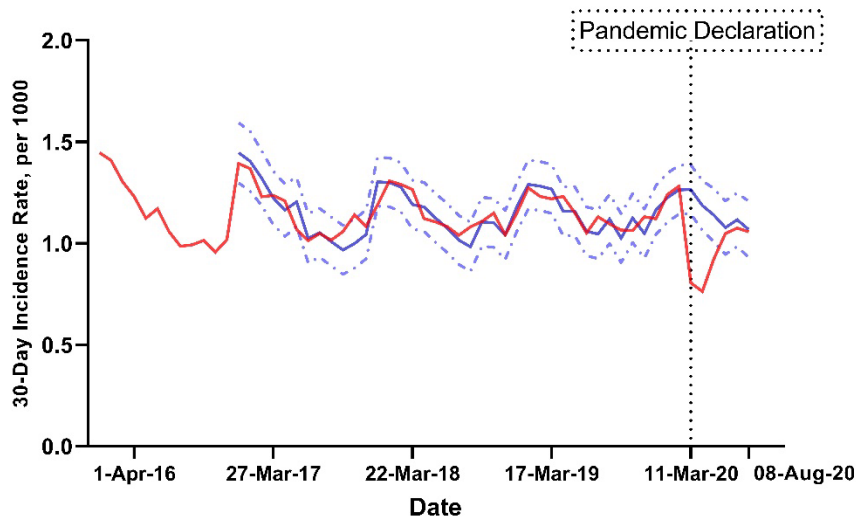

Supplement: S2 Fig — The upper panel shows trends in new atrial fibrillation diagnoses per 1000 female individuals for 30-day intervals, from 01/02/2016 to 09/06/2020. The lower panel shows trends in new atrial fibrillation diagnoses per 1000 male individuals for 30-day intervals, from 01/02/2016 to 09/06/2020. Solid red lines represent observed values. Solid blue lines represent predictions from ARIMA models the absence of the COVID-19 pandemic. In other words, they represent trends in AF diagnosis that would have been observed if there had not been a change in AF diagnoses following the COVID-19 pandemic. Dashed blue lines represent confidence intervals. (PDF) [file pone.0281068.s006.pdf]
